# Supplementary material for: Aqueous habitats and carbon inputs shape the microscale geography and interaction ranges of soil bacteria
Source: Commun Biol. 2023 Mar 25;6:322. doi: 10.1038/s42003-023-04703-7 (PMC10039866; doi:10.1038/s42003-023-04703-7)
Supplement: Supplementary file 2 — Supplementary Information [file 42003_2023_4703_MOESM2_ESM.pdf]

Supplementary Information for “Aqueous habitats and carbon inputs shape the microscale geography and interaction ranges of soil bacteria”

Authors: Samuel Bickel[1,2]* (0000-0002-9839-4591) and Dani Or[1,3] (0000-0002-3236-2933)

This document contains:

- Supplementary Notes: S1-S2
- Supplementary Figures: S1-S6
- Supplementary References

Supplementary Notes

Note S1. How aqueous diffusion shapes bacterial interactions in unsaturated soil

The reduction of liquid connectivity and thus bulk aqueous diffusivity (D_0 ≈ 10^{-10} m^2 s^{-1}) in unsaturated soils can be represented by an effective diffusivity function $D_e(\theta)$ that varies with soil water content θ and total porosity[1] θ_s (Equation 8). Variability in D_0 for different substances are not considered here given the governing role of soil moisture content for the disproportional reduction of D_e in unsaturated soils. We postulate that the consideration of diffusion distances for a given time t ($L_D = \sqrt{D_e t}$), and diffusion times for a given bacterial cell cluster separation distance L ($t_D = L^2/D_e$), offer quantitative measures that link soil bacterial micro-geography with ecological interaction potential for different soils and climatic conditions. In a fertile soil in temperate regions with a high bacterial cell density of 10^{12} g^{-1}_{soil} and a specific surface area of 100 m^2 g^{-1}_{soil} (loamy soil), we expect up to 10^4 cells per mm^2 grain surface area. Assuming that bacterial cells are distributed in colonies of 100 cells each (representing local cell division), the average separation distance (for uniform distribution) is about 100 µm, which will support interactions between neighboring colonies under wet conditions by diffusion of metabolites via the aqueous phase (with L_D = 800 µm per day using θ = 0.3, θ_s = 0.5, and D_e ≈ $10^{-2}D_0$; Equation 8). In contrast, for soils in drier climates with naturally lower cell density of 10^2 cells per mm^2 (10^8 g^{-1}_{soil} in sandy soils with 1 m^2 g^{-1}_{soil}), bacterial colonies would be separated by millimetric gaps with timescales for substrate diffusion across this distance of the order of years (with θ = 0.05, and $D_e(\theta)$ ≈ $10^{-4}D_0$).

Note S2. Bacterial interaction heuristic model (BIHM) parametrization based on simulated cell clusters

The power law that characterizes cell cluster size distributions is compatible with a variety of processes ranging from collective motion[2,3] to growth models with preferential attachment (e.g., diffusion-limited aggregation[4]) and is sensitive to spatial constraints[2,4]. It was previously used to characterize the distribution of pathogenic bacteria on leaf surfaces[5]. Here, the sizes of the largest cluster are bounded and depend on the total number of individuals, for example, prescribed by carrying capacity or bulk cell density ρ_c. For infinite carrying capacity ($n_c \to \infty$) the relation only depends on b that is expected to change with spatial constraints[2]. In the case where b = 1, the distribution converges to the log-series[6].

Variations in cell density are associated with changes in the distribution of bacterial cluster sizes, that can thus be deduced from macroscopic quantities (e.g., carbon input and water content). The relations of n_c and b with cell density (Equations 19 and 20) obtained from the spatially explicit individual based model (SIM) were used to parametrize the BIHM (Figure S4). Overall, the modeled parameters differed from values expected from complete spatial randomness (i.e., a homogeneous spatial Poisson process) under varying cell densities (Figure S4 a and c). Particularly, a larger n_c suggested bigger cell clusters at lower cell densities compared to randomly generated distributions. Results of the microcosm experiments could not be used for model parametrization as the variation in cell densities was too small (no treatment effect due to high nutrient background of the soil). Results from an independent study[7] were not included because the exponents could not be estimated reliably with the small number of cell clusters analyzed (and parameter estimation is uncertain[2] for small n_c).

Overall, the size of the largest bacterial cell cluster increased with the total number of cells analyzed for both experiments and the SIM (Figure S1 a). Similar patterns have been reported for cell clusters on leaf surfaces where the size of the largest clusters depended on humidity[5]. In our microcosm experiment, the proportion of cells in the largest cluster did not change substantially across treatments for both timepoints of observation (Figure S1 b). Similarly, the proportion of spatially isolated, individual cells did also not change considerably (Figure S1 c). The minor differences in cell cluster size distributions observed in our microcosm experiments correspond to the minor changes in cell densities (Figure S1 d). This allowed for pooling the cell cluster size distributions (Fig. 4 d).

Supplementary Figures

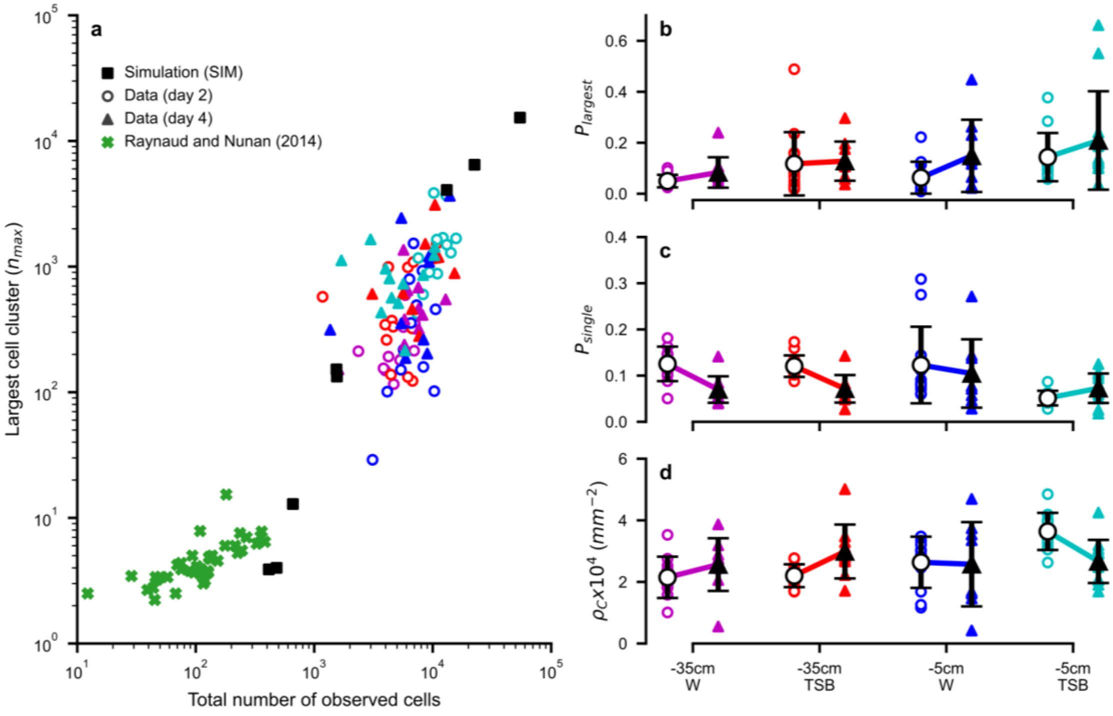

Figure S1. Largest bacterial cell clusters increase with cell density. a, The size of the largest cell cluster increases consistently with the total number of observed cells for microcosm surface imaging data, results of the spatially explicit individual-based model (SIM), and thin-section imaging data from an independent study[7]. **b**, The proportion of cells in the largest cluster ($P_{largest}$) across nutrient and hydration conditions of the microcosm experiment. **c**, The proportion of isolated, single cells (P_{single}) and **d**, Cell densities across experimental treatments. The hydration condition (matric potential) and the nutrient conditions (water W and tryptic soy broth TSB) that were applied in the experiment are indicated.

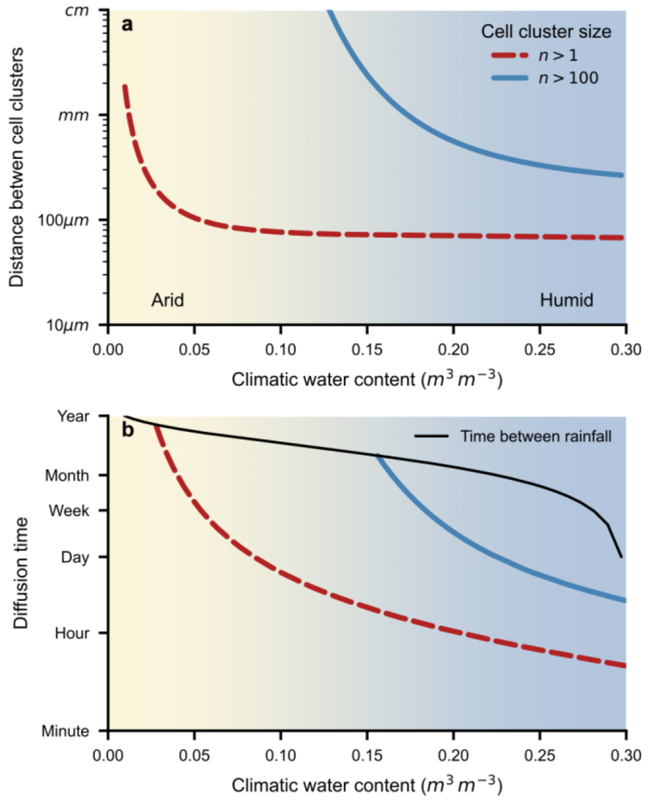

Figure S2. Bacterial cell cluster size variations constrain soil bacterial interactions. a, The modeled distance between cell clusters as a function of climatic water content for two lower bounds on cluster sizes n. The average distances between clusters with two or more cells and between clusters with more than 100 cells are shown in red and blue, respectively. **b**, The time needed for a small molecule to diffuse across the average distance between clusters. The average time between rainfall is shown as an upper bound (black line).

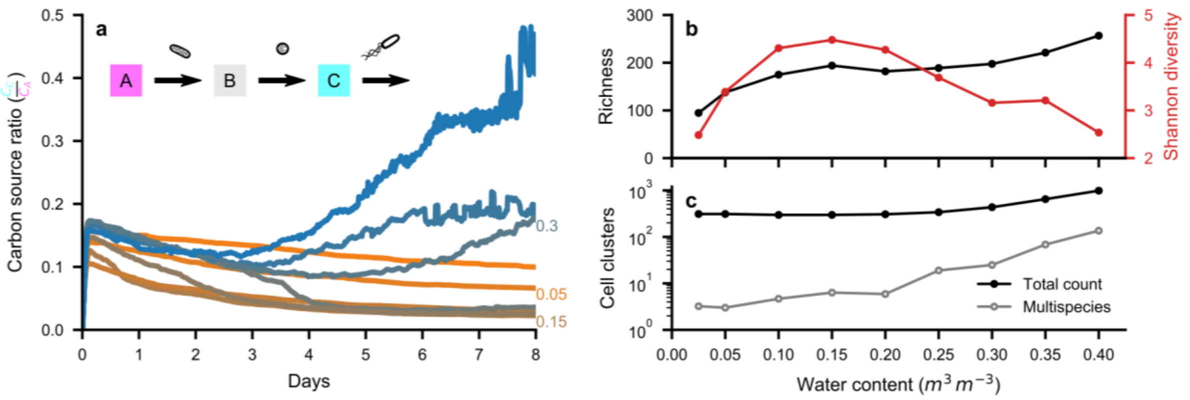

Figure S3. Enhanced metabolic interactions in wet soils with implications for species diversity. a, Dynamics of carbon source concentrations (median, n = 9) as obtained from the spatially explicit individual-based model (SIM). The SIM considers a degradation pathway from carbon source A to C (consumption of A releases B to the aqueous phase, etc.). The concentration ratio of the product C (cyan) to the source compound A (magenta) indicates interactions via diffusion in the aqueous phase (C_C/C_A, colored lines from orange to blue indicate different water contents in m^3m^{-3}). **b,** The bacterial diversity changes with hydration conditions. Richness (black) increases with increasing water content. Shannon diversity (red) decreases towards wet conditions indicating reduced evenness. **c**, The number of cell clusters (black) and the number of multispecies cell clusters (grey) increase with water content.

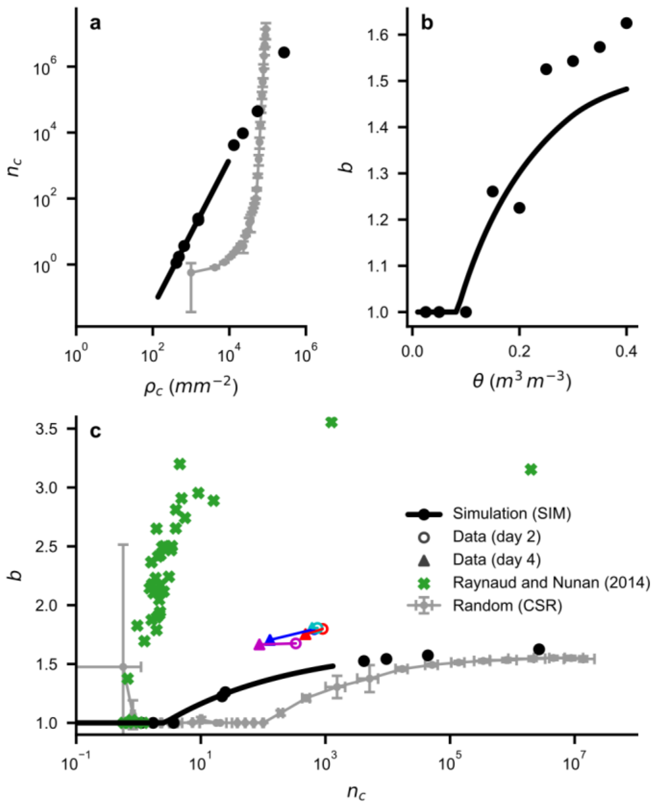

Figure S4. Modeled cell cluster size distribution parameters. a-c, A power law with an exponential cutoff, is used to describe the soil bacterial cell cluster size distribution. Estimated parameters are shown for the spatially explicit individual-based model (SIM, black symbols) with parametrization for typical cell densities (black solid line). **a**, For comparison, parameters were also calculated for complete spatial randomness under varying cell density (grey symbols; mean ± SD, n = 24). Cutoff parameter n_c is related to cell density ρ_c. **b**, Exponent b is not independent of n_c and varies with water content θ mediated by ρ_c. **c**, The relation between n_c and b is used for model parametrization. Parameters are shown for data from microcosms, an independent study[7], and the SIM.

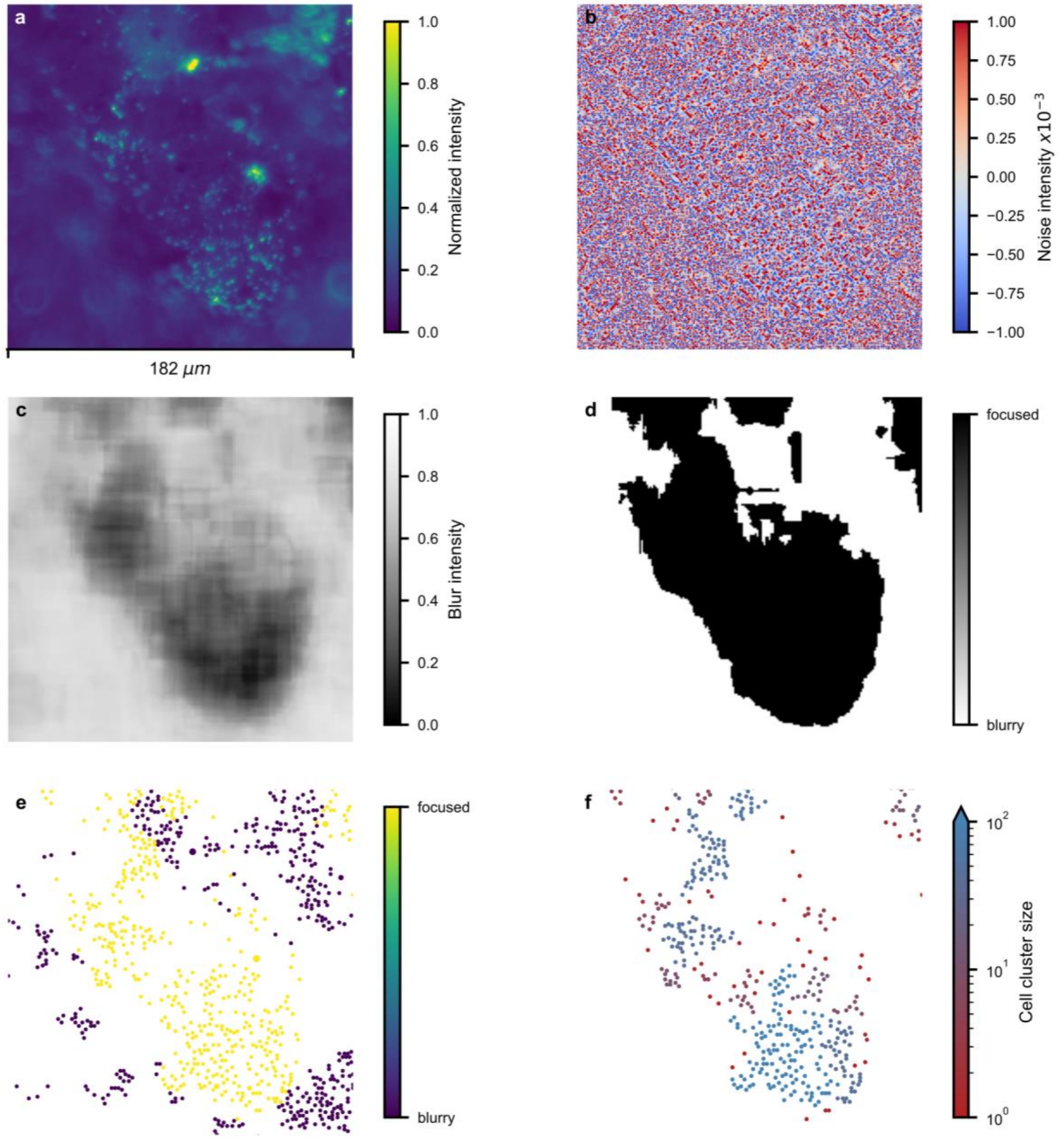

Figure S5. **Image processing, cell detection and cell clustering. a**, Normalized and denoised fluorescence intensity of SYTO9 is shown for an image section of the microcosm experiment at one-micrometer resolution. The size of the image section is indicated. **b**, Noise that was removed from the image. **c**, Blur map. **d**, quantification of the soil surface area that lies in the focal plane. **e**, The detected cells are labelled if they lie in the focal area (highlighted) and are used for cell density estimation. **f**, Cells are clustered with their neighbors within 5 µm to estimate cluster sizes (color).

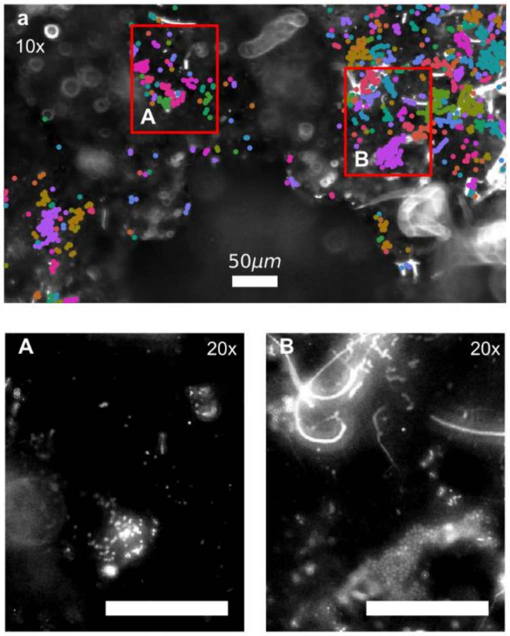

Figure S6. **Soil bacterial cell clusters observed in the soil microcosm experiment. a**, Cell clusters detected after two days of growth stained with SYTO9 (greyscale) and imaged at one-micrometer resolution (10x). Cells are grouped into the same cluster if they are within five micrometers (shown as distinct colors for each group). Only cells in the focal plane are labeled and used in the analysis. Detailed view of soil micro-colonies (boxes **A** and **B**) at 0.5-micrometer resolution (20x); truncated below the median and above 99 percent intensity for visualization. (**A**) Small settlement of round cells on a soil grain with large variation in surface topology. (**B**) Filamentous growth is detected as "chains" of individual cells. Densely packed colonies of round cells with low fluorescence intensity are visible in the lower half.

Supplementary References

1. Millington, R. J. & Quirk, J. P. Permeability of porous solids. *Trans. Faraday Soc.* **57**, 1200–1207 (1961).
2. Bonabeau, E., Dagorn, L. & Fréon, P. Scaling in animal group-size distributions. *Proc. Natl. Acad. Sci.* **96**, 4472–4477 (1999).
3. Zhang, H. P., Be'er, A., Florin, E.-L. & Swinney, H. L. Collective motion and density fluctuations in bacterial colonies. *Proc. Natl. Acad. Sci.* **107**, 13626–13630 (2010).
4. Nicolás-Carlock, J. R. & Carrillo-Estrada, J. L. A universal dimensionality function for the fractal dimensions of Laplacian growth. *Sci. Rep.* **9**, 1120 (2019).
5. Monier, J.-M. & Lindow, S. E. Frequency, Size, and Localization of Bacterial Aggregates on Bean Leaf Surfaces. *Appl. Environ. Microbiol.* **70**, 346–355 (2004).
6. Pueyo, S., He, F. & Zillio, T. The maximum entropy formalism and the idiosyncratic theory of biodiversity. *Ecol. Lett.* **10**, 1017–1028 (2007).
7. Raynaud, X. & Nunan, N. Spatial Ecology of Bacteria at the Microscale in Soil. *PLoS ONE* **9**, e87217 (2014).
