# Supplementary material for: Aqueous habitats and carbon inputs shape the microscale geography and interaction ranges of soil bacteria
Source: Commun Biol. 2023 Mar 25;6:322. doi: 10.1038/s42003-023-04703-7 (PMC10039866; doi:10.1038/s42003-023-04703-7)
Supplement: Supplementary file 5 — Reporting Summary [file 42003_2023_4703_MOESM5_ESM.pdf]

nature portfolio

Corresponding author(s): Samuel Bickel

Last updated by author(s): Feb 23, 2023

Reporting Summary

Nature Portfolio wishes to improve the reproducibility of the work that we publish. This form provides structure for consistency and transparency in reporting. For further information on Nature Portfolio policies, see our Editorial Policies and the Editorial Policy Checklist.

Statistics

For all statistical analyses, confirm that the following items are present in the figure legend, table legend, main text, or Methods section.

n/a	Confirmed	
☐	☒	The exact sample size (*n*) for each experimental group/condition, given as a discrete number and unit of measurement
☐	☒	A statement on whether measurements were taken from distinct samples or whether the same sample was measured repeatedly
☒	☐	The statistical test(s) used AND whether they are one- or two-sided<br>*Only common tests should be described solely by name; describe more complex techniques in the Methods section.*
☒	☐	A description of all covariates tested
☐	☒	A description of any assumptions or corrections, such as tests of normality and adjustment for multiple comparisons
☐	☒	A full description of the statistical parameters including central tendency (e.g. means) or other basic estimates (e.g. regression coefficient) AND variation (e.g. standard deviation) or associated estimates of uncertainty (e.g. confidence intervals)
☒	☐	For null hypothesis testing, the test statistic (e.g. *F*, *t*, *r*) with confidence intervals, effect sizes, degrees of freedom and *P* value noted<br>*Give P values as exact values whenever suitable.*
☒	☐	For Bayesian analysis, information on the choice of priors and Markov chain Monte Carlo settings
☒	☐	For hierarchical and complex designs, identification of the appropriate level for tests and full reporting of outcomes
☐	☒	Estimates of effect sizes (e.g. Cohen's *d*, Pearson's *r*), indicating how they were calculated

Our web collection on statistics for biologists contains articles on many of the points above.

Software and code

Policy information about availability of computer code

Data collection	For microscopic imaging the EVOS FL Auto Cell Imaging System Software (REV 32044) was used. Python (3.9) was used with SciPy (1.8.1), skimage (0.18.0). Collected data and custom code are available on Zenodo (10.5281/zenodo.6325132).
Data analysis	Python (3.9) was used with SciPy (1.8.1), skimage (0.18.0), powerlaw (1.5) and HDBSCAN (0.8.28). Custom code and generated data are available on Zenodo (10.5281/zenodo.6325132).

For manuscripts utilizing custom algorithms or software that are central to the research but not yet described in published literature, software must be made available to editors and reviewers. We strongly encourage code deposition in a community repository (e.g. GitHub). See the Nature Portfolio guidelines for submitting code & software for further information.

Data

Policy information about availability of data

All manuscripts must include a data availability statement. This statement should provide the following information, where applicable:

- Accession codes, unique identifiers, or web links for publicly available datasets
- A description of any restrictions on data availability
- For clinical datasets or third party data, please ensure that the statement adheres to our policy

All maps generated, the image data and the image analysis code are archived on Zenodo (DOI: 10.5281/zenodo.6325132). The code underlying the SIM was previously published (DOI: 10.5281/zenodo.3558542).

Human research participants

Policy information about studies involving human research participants and Sex and Gender in Research.

Reporting on sex and gender	not applicable
Population characteristics	not applicable
Recruitment	not applicable
Ethics oversight	not applicable

Note that full information on the approval of the study protocol must also be provided in the manuscript.

Field-specific reporting

Please select the one below that is the best fit for your research. If you are not sure, read the appropriate sections before making your selection.

☒ Life sciences ☐ Behavioural & social sciences ☐ Ecological, evolutionary & environmental sciences

For a reference copy of the document with all sections, see nature.com/documents/nr-reporting-summary-flat.pdf

Life sciences study design

All studies must disclose on these points even when the disclosure is negative.

Sample size	No calculations to determine sample size were performed as the study does not rely on statistical testing (i.e., models were not fitted and mechanistic modelling was used for prediction).
Data exclusions	No data was excluded from the analysis.
Replication	Mechanistic modelling was performed and did not rely on information collected during the study (i.e., the model and assumptions were defined a priori). In the mechanistic model experiments replication was performed until stable results (ensemble averages) were obtained.
Randomization	Randomization was not required as all samples within a group were subject to the same conditions and no boundary/neighbourhood effects were observed. Replicate soil samples were subject to constant hydration and nutrient conditions at the scale relevant for measurement. Additionally, the same soil was subject to different conditions and groups were assigned after the soil sample was distributed in the microcosms (i.e., no information on the treatment was available when microcosms were set up).
Blinding	Blinding was not relevant for the study and treatements were applied after samples were distributed. Microcosms with soil samples were prepared before treatments were assigned.

Reporting for specific materials, systems and methods

We require information from authors about some types of materials, experimental systems and methods used in many studies. Here, indicate whether each material, system or method listed is relevant to your study. If you are not sure if a list item applies to your research, read the appropriate section before selecting a response.

Materials & experimental systems

n/a	Involved in the study	
☒	☐	Antibodies
☒	☐	Eukaryotic cell lines
☒	☐	Palaeontology and archaeology
☒	☐	Animals and other organisms
☒	☐	Clinical data
☒	☐	Dual use research of concern

Methods

n/a	Involved in the study	
☒	☐	ChIP-seq
☒	☐	Flow cytometry
☒	☐	MRI-based neuroimaging
